# Population Genetics Reveals That the Western Tianshan Mountains Populations of *Agrilus mali* (Coleoptera: Buprestidae) May Have Not been Recently Introduced

**DOI:** 10.3389/fgene.2022.857866

**Published:** 2022-03-24

**Authors:** Huiquan Sun, Feiran Jia, Wenxia Zhao, Zhongfu Zhou, Chengjin Li, Jianjun Wang, Yanxia Yao

**Affiliations:** ^1^ Key Laboratory of Forest Protection of National Forestry and Grassland Administration, Ecology and Nature Conservation Institute, Chinese Academy of Forestry, Beijing, China; ^2^ Liaoning Academy of Forest Science, Shenyang, China

**Keywords:** buprestidae, mitochondrial DNA, genetic differentiation, genetic structure, geographical population

## Abstract

*Agrilus mali* Matsumura is a wood-boring beetle that aggressively attacks species of the genus *Malus,* that has recently caused serious damage to the wild apple tree *M*. *sieversii* (Lebed.) in the western Tianshan Mountains in Xinjiang. It was first detected there in the early 1990s and spread rapidly, being thus considered a regional invasive pest. To explore the possible outbreak mechanism of the local population and characterize the genetic differentiation of *A. mali* across different regions of China, we used three mitochondrial genes (*COI*, *COII*, and *CytB*) to investigate the genetic diversity and genetic structure of 17 *A. mali* populations containing 205 individuals collected from five Chinese provinces. Among them, nine populations were from the western Tianshan Mountains. Ultimately, of the 136 pairwise *F*_*st*_ comparisons, 99 showed high genetic differentiation among overall populations, and Tianshan populations exhibited significant differentiation with most of the non-Tianshan populations. Furthermore, *A. mali* populations represented relatively abundant haplotypes (54 haplotypes). Nine populations from the Tianshan Mountains showed 32 haplotypes (26 of which were unique), displaying relatively high genetic diversity. Additionally, the Mantel test revealed population genetic differentiation among either overall populations or the Tianshan Mountains populations, likely caused by geographical isolation. Phylogenic relationships showed that all populations clustered into three clades, and Tianshan Mountains populations, including CY, occupied one of the three clades. These results suggest that *A. mali* in the western Tianshan region has possibly been present in the area for a long period, and may not have been introduced recently. Highly frequent gene flows within Tianshan populations are possibly caused by human activities and may enhance the adaptability of *A. mali* along the western Tianshan Mountains, leading to periodic outbreaks. These findings enhance our understanding of jewel beetle population genetics and provide valuable information for pest management.

## Introduction

*Agrilus mali* Matsumura (Coleoptera: Buprestidae) is a serious wood-boring beetle and considered to originate in Northeast Asia, including North Korea, the Russian Far East, and Eastern China (Chebanov, 1977; Nikritin, 1994; EPPO, 2021). In recent decades, the beetle has severely endangered the wild apple *Malus sieversii* (Lebed.), in the wild fruit forests of the Tianshan Mountains, Xinjiang, Western China (Cui et al., 2018). The host apple is an important germplasm gene resource and an ancestor of cultivated apples (Richards et al., 2009). In the early 1990s, this beetle broke out in the wild fruit forests of the western Tianshan Mountains. It was thought that the introduction of apple seedlings from Northeast China or the Shandong Province caused the invasion of *A. mali* (Ji et al., 2004; Bozorov et al., 2019; Zhang et al., 2021). As the beginning of the invasion was overlooked, the population source and the exact spreading mechanisms remain unclear. However, studying its population genetics can provide definite insights into its origin and subsequent spread.

Populations are basic units of biological evolution, and the study of population genetics is key in understanding biological genetic diversity and species evolution (Frankham et al., 2004). Population genetics can provide evidence of historical biogeographic events (Avise, 2000) and reveal significant information on population dynamics (Gupta and Preet, 2014). Population genetics can also help reconstruct the demographic history and understand whether a population is native or invasive (Zheng et al., 2013; Men et al., 2017). Population genetics also helps determine whether an invasion was the result of multiple independent introduction events or a long-term spread from a single source population (Xun et al., 2016; Zhang et al., 2020; Du et al., 2021). In this case, genetic polymorphisms transmitted in a strictly Mendelian manner provide useful information. Thus, the use of available markers is crucial (Cavalli-Sforza, 1998). With the development of molecular biology techniques, an increasing number of molecular markers have been used in the study of population genetics, such as mitochondrial DNA, ribosomal DNA, and microsatellite sequences (Bray et al., 2011; Lopez et al., 2014; Zhang et al., 2015; Yao et al., 2020).

Previously, eight populations (seven from Tianshan and one from Liaoning) of this beetle were investigated genetically based on seven simple sequence repeat (SSR) markers, with the Tianshan populations revealing high genetic similarity and showing significant differentiation when compared with the Liaoning population (Fang et al., 2017). Owing to a lack of sufficient data, the genetic structure of this beetle population is still unclear.

mtDNA plays an important role as a basic tool in insect ecology and genetic studies (Dong et al., 2021). According to Zardoya and Meyer (1996), the mitochondrial DNA genes, cytochrome oxidase subunit I (*COI*), cytochrome oxidase subunit II (*COII*), and cytochrome b (*CytB*) are ideal molecular markers for evolutionary analysis. In the present study, the geographical pattern of genetic variation for this species was assessed using the above three mtDNA genes (*COI*, *COII*, and *CytB*) from 17 populations across China. The main purposes were to (i) analyze the genetic structure of *A. mali*, (ii) infer the demographic history of *A. mali*, and (iii) discuss the genetic status of populations in the western Tianshan Mountains. By comparing the genetic structure of populations from different habitats, we attempted to understand the relationships between populations and evaluate factors that may affect genetic variation. Information on the genetic diversity of *A. mali* populations may increase our knowledge of these beetle populations and enhance their effective control.

## Materials and Methods

### Specimen Collection and DNA Extraction

**FIGURE 1:**
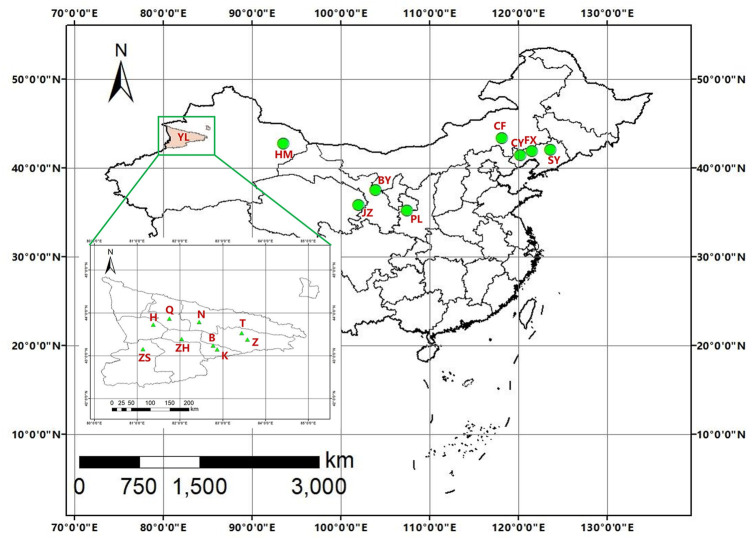
Geographical distribution of 17 sampling populations of *A. mali*.

The damaged branches of host trees were collected in wild fruits forests and orchards from May to August 2018 to 2021. Branches were then cultured *in vitro* at room temperature. Once adult beetles emerged, they were placed into the centrifugal tubes and stored at −80°C prior to DNA extraction. Genomic DNA was extracted from an individual without the abdomen and elytra using a TIANamp Genomic DNA Kit (TIANGEN, Beijing, China) according to the manufacturer’s instructions. Voucher specimens were deposited at the insect museum of the Chinese Academy of Forestry, Beijing, China. Hundreds of *A. mali* adult individuals were obtained from 17 locations in China (Table 1; Figure 1).

**TABLE 1:** Sampling information of 17 geographical populations of *A. mali* in China.

Province	Locality	Code	Sample size	Latitude (°N)	Longitude (°E)	Date	Sample area category
Liaoning	Fuxin city	FX	12	41°55′55″	121°33′4″	June 2020	Plantation
	Chaoyang city	CY	13	41°29′52″	120°13′29″	June 2021	Plantation
	Shenyang city	SY	10	42°4′7″	123°37′38″	June 2019	Plantation
Inner Mongolia	Chifeng city	CF	11	43°23′20″	118°8′17″	May 2021	Plantation
Gansu	Pingliang city	PL	13	35°17′16″	107°29′40″	June 2021	Plantation
	Baiyin city	BY	4	37°33′47″	103°53′15″	June 2019	Plantation
Qinghai	Jianzha County	JZ	10	35°52′46″	102°1′31″	July 2020	Plantation
Xinjiang Uygur	Hami city	HM	3	42°49′6″	93°30′55″	June 2018	Plantation
	Yining County, Qingnian farm	YLQ^a^	19	43°52′15″	81°45′1″	July 2019	Plantation
	Zhaosu County	YLZS^a^	15	43°9′20″	81°7′57″	June 2020	Natural forest
	Nileke County	YLN^a^	12	43°47′18″	82°26′55″	July 2019	Plantation
	Hainuke town	YLH^a^	13	43°43′41″	81°22′34″	May 2019	Plantation
	Gongliu County, Zonghe farm	YLZH^a^	12	43°23′24″	82°2′24″	June 2019	Plantation
	Gongliu county, Kuerdening	YLK^a^	17	43°9′6″	82°52′10″	July 2019	Natural forest
	Balian	YLB^a^	13	43°14′24″	82°46′12″	July 2019	Natural forest
	Xinyuan County, Tuergen town	YLT^a^	18	43°31′51″	83°26′26″	July 2019	Natural forest
	Xinyuan County, Zhiwuyaun	YLZ^a^	10	43°22′48″	83°34′30″	July 2019	Natural forest

a Sampling locations are all in the western Tianshan Mountains, and were grouped as YL.

### PCR Amplification and Sequencing

Three mitochondrial protein-coding genes (*COI*, *COII*, and *CytB*) were utilized as molecular markers in the present study. Based on the published mitochondrial genome sequence of *A. mali* (Sun et al., 2020), we designed specific primers for the amplification of complete *COI*, *COII*, and *CytB* sequences using the software Primer Premier version 5 (Lalitha, 2000). Details of the primer sequences and product sizes are provided in Supplementary Table S1.

Polymerase Chain Reaction (PCR) amplification was conducted in a total volume of 25 μl containing 1.5 μl genomic DNA, 1 μl each of forward and reverse primers (10 μmol/L), 12.5 μl 2× Flash PCR MasterMix (CWBIO, China), and 9 μl ddH_2_O. PCR amplification was performed with an initial denaturation at 98°C for 1 min; 35 cycles of 10 s at 94°C, 15 s at a primer-specific annealing temperature (46°C for *COI*, 49°C for *COII*, and 53°C for *CytB*), and extension at 72°C for 30 s; and a final elongation at 72°C for 1 min. The PCR products were visualized on 1.0% agarose gels under UV light. The sequencing reaction was carried out using an ABI BigDye Terminator version 3.1 cycle sequencing kit on an ABI 3730XL (Tsingke Biotechnology Co., Ltd., Beijing, China) for Sanger sequencing in both directions.

### Sequence Alignment and Genetic Diversity Inferences of the Data Sets

To confirm that the correct sequences were obtained, we compared each sequence with the non-redundant (nr) database of National Center for Biotechnology Information (NCBI) using BLASTN. Three mitochondrial gene sequences were analyzed and edited using Geneious R11.1.5 (https://www.geneious.com) (Kearse et al., 2012) and MEGA version 11 (Tamura et al., 2021). Deletions or insertions in the nucleotide sequences of mitochondrial protein-coding genes (*COI*, *COII*, and *CytB*) were inferred from the amino acid alignment. The sequence of each PCG (excluding stop codons) was then aligned individually as per codon-based multiple alignments using the MAFFT algorithm, which was implemented in the TranslatorX online platform (Abascal et al., 2010). Subsequently, the three genes of an individual were concatenated using MEGA version 11 to generate a combined sequence of 2,913 bp as a single locus because of the lack of recombination of mtDNA (Moraes et al., 2002). The nucleotide composition and the number of variable sites, conserved sites, parsimony informative sites, and singleton variable sites of the three single partial gene sequences, as well as of the combined sequences were analyzed using MEGA version 11.

Genetic diversity was estimated by the number of polymorphic sites (S), the number of haplotypes (h), haplotype diversity (Hd), average number of nucleotide differences (K), and nucleotide diversity (π) for concatenated genes, using DnaSP version 5.0 (Librado and Rozas, 2009) or Arlequin version 3.5 (Excoffier and Lischer, 2010). Besides the analysis of each Tianshan population alone, we combined the nine populations in these regions as a single group (YL) to examine their genetic diversity for comparison with non-Tianshan populations. For the comparisons, we additionally divided the Tianshan populations into natural forest populations (YLNA) and plantation populations (YLPL) (Table 3).

The haplotype sequences for each gene were exported by DnaSP version 5.0. The haplotype sequences were deposited in GenBank under the accession numbers OM177028–OM177057 for *COI*, and OM212329–OM212346 for *COII*. All *CytB* sequences were also deposited in GenBank under the accession numbers OM264916–OM265120, and haplotype sequences are available in the Zenodo database with a DOI of https://doi.org/10.5281/zenodo.6107571.

### Genetic Differentiation and Genetic Flow

Based on combined genes sequences, pairwise fixation (*F*_*st*_) and gene flow indices (*N*_*m*_) were estimated using the DnaSP version 5.0 (Librado and Rozas, 2009). With the criterion for genetic differentiation set by Wright (1984), the genetic differentiation is low for *F*_*st*_ < 0.05, moderate for 0.05 < *F*_*st*_ < 0.15, high for 0.15 < *F*_*st*_ < 0.25, and very high for *F*_*st*_ > 0.25 (Govindaraju, 1989). Similarly, genetic flow was low for *N*_*m*_ < 1, high for 1 < *N*_*m*_ < 4, and very high for *N*_*m*_ > 4 (Boivin et al., 2004).

The analysis of molecular variance (AMOVA) based on *F*_*st*_ (using haplotype frequencies) was performed using 10,000 permutations to estimate the hierarchical genetic structure using Arlequin version 3.5 (Excoffier and Lischer, 2010). Two hierarchical levels, among populations and within populations, were defined.

To test isolation by distance (IBD), the pairwise genetic distance and geographical distance (km) matrices between all 17 sampled populations were compared using the Mantel test with 9,999 permutations (Mantel, 1967). Pairwise average genetic distances between different populations were calculated using the Kimura-2-parameter model in MEGA version 11 (Tamura et al., 2021). Geographical distances were calculated from longitude and latitude data using the Geographic Distance Matrix Generator version 1.2.3 (Ersts, 2018). Mantel analysis was performed using GenAleX version 6.5 (Peakall and Smouse, 2012).

### Phylogenetic Analysis and Network Construction

The dataset of mitochondrial haplotypes was analyzed under Bayesian inference (BI) using MrBayes version 3.2.5 (Ronquist and Huelsenbeck, 2003). The congeneric species of *Agrilus planipennis* were used as an outgroup. For Bayesian analyses, we analyzed the combined genes based on the GTR + I + G model, which was selected using jModelTest version 2.1.7 (Posada, 2008). Then, two simultaneous runs of 10,000,000 generations were conducted for the matrix, each of which was sampled every 1,000 generations with a burn-in of 25%. Trees inferred prior to stationarity were discarded as burn-in, and the remaining trees were used to construct a 50% majority-rule consensus tree. The relationships among haplotypes were further tested by creating a median-joining algorithm using PopART version 1.7 (Leigh and Bryant, 2015).

### Demographic History

To explore the demographic history of different lineages, neutral tests using Tajima’s D (Tajima, 1989) and Fu’s Fs (Fu, 1997) for the defined populations were performed using DnaSP version 5.0 (Librado and Rozas, 2009), with significantly negative values indicating population expansion and significantly positive values indicating processes such as population subdivision or recent population bottlenecks (Kimura, 1983; Tajima, 1989).

Additionally, the distribution of pairwise differences (mismatch distributions) between each pair of sequences was analyzed using DnaSP version 5.0 (Librado and Rozas, 2009) with default parameters to detect population expansion through a linear fitting relationship between the observed and simulated curves and infer demographic equilibrium or expansion according to Rogers and Harpending (1992).

## Results

### Sequence Analyses and Genetic Diversity

The final combined mitochondrial dataset included 2,913 bp of protein-coding regions (*COI*:1,395 bp, *COII*:582 bp, *CytB*:936 bp). No insertions or deletions were detected in these fragments. To ensure the credibility of the data, we deleted a 186 bp sequence containing the poly-T base in the *CytB* gene. The nucleotide compositions of the 17 geographical populations were consistent. The obtained combined sequences showed base compositions similar to those reported previously in mitochondrial sequences for other insects, with a heavy bias towards A/T (68.7%), which was in accordance with the mitochondrial nucleotide composition (Jermiin and Crozier, 1994).

Every mitochondrial gene displayed several haplotypes, with a total of 30 for *COI*, 18 for *COII*, and 37 for *CytB*, among which 7, 5, and 24 were shared, respectively. Details of the haplotype distribution of mitochondrial *COI*, *COII*, *CytB*, and their combined genes in each *A. mali* population are shown in Supplementary Tables S3–S6. Among the combined genes, 151 polymorphic sites (including 134 parsimony sites and 17 singleton sites) and 54 haplotypes (including 37 unique haplotypes) were found among all samples (Tables 2, 3). For a single population, the average number of haplotypes (h) ranged from 2 (HM) to 13 (YLK); the average number of nucleotide differences (K) ranged from 0.667 (HM) to 28.974 (CY), with an average of 10.933 (Table 3); haplotype diversity (Hd) within populations ranged from 0.590 (PL) to 0.963 (YLK), with an average of 0.793; and nucleotide diversity (π) ranged from 0.00023 (HM) to 0.00955 (CY). The whole Chinese population exhibited high haplotype diversity and high nucleotide diversity (Hd = 0.924; *π* = 0.00752).

**TABLE 2:** Variation of *COI*, *COII*, *CytB* and combined genes of 17 populations of *A. mali*.

Genes	Sequences length (bp)	T (%)	C (%)	A (%)	G (%)	A + T (%)	Cs	Vs.	*p*	S
*COI*	1,395	38.2	16.8	28.6	16.4	66.8	1,324	71	58	13
*COII*	582	40.7	16.7	30.3	12.3	71.0	556	26	24	2
*CytB*	936	38.7	16.8	31.3	13.2	68.5	882	54	52	2
Combined	2,913	38.9	16.8	29.8	14.6	68.7	2,762	151	134	17

Cs, conserved sites; Vs, variable sites; P, parsimony informative sites; S, singleton variable sites.

**TABLE 3:** Genetic diversity parameters and neutrality tests among 17 populations of *A. mali* based on combined genes.

Sample code	Combined					Neutrality test and significance test	
	S	h	Hd	K	π	Tajima’s D	Fu’s fs
BY	24	3	0.833	15.833	0.00546	2.15657	3.414
CF	64	5	0.782	17.855	0.00613	−0.86918	6.753
CY	65	4	0.769	28.974	0.00955	1.72964	14.628
FX	4	4	0.600	1.164	0.00040	−0.54169	−0.419
HM	1	2	0.667	0.667	0.00023	—	—
JZ	28	4	0.733	11.267	0.00387	0.66214	6.088
PL	29	3	0.590	8.282	0.00284	−0.49809	8.704
SY	13	5	0.800	5.911	0.00203	1.30295	2.020
YLB(NA)	30	7	0.846	12.154	0.00417	1.12810	2.944
YLH(PL)	13	6	0.833	6.615	0.00227	2.38509*	2.131
YLK(NA)	33	13	0.963	11.588	0.00398	0.76656	−1.547
YLN(PL)	11	3	0.591	5.015	0.00172	1.56790	5.613
YLQ (PL)	36	11	0.912	8.795	0.00302	−0.58552	−0.023
YLT (NA)	34	11	0.889	7.268	0.00250	−1.07113	−0.903
YLZ (NA)	22	8	0.933	8.556	0.00294	0.47350	−0.657
YLZH(PL)	28	4	0.742	12.394	0.00425	1.50954	7.934
YLZS(NA)	31	6	0.800	13.314	0.00457	1.67178	5.923
YLNA	48	27	0.881	10.740	0.00369	0.21516	−1.894
YLPL	36	16	0.822	8.951	0.00307	0.47249	1.994
YL	53	32	0.862	10.157	0.00349	0.06834	−1.800
Total	151	54	0.924	21.912	0.00752	−0.52157	−0.541

For sample code information for each population, see Table 1. YL, represents the sum of populations in the Western Tianshan Mountains. YLNA, and YLPL, represent the sum of natural forests and plantation populations in this region, respectively. S, variable sites, h, number of haplotypes, Hd, haplotype diversity; K, average nucleotide difference, *π*, nucleotide diversity. In two neutrality tests, “*” rep-resents statistical significance (*p* < 0.05). Insignificant values (0.05 < *p* < 0.10 or *p* > 0.10) are not labeled. “—” represents that no polymorphisms in sequences were provided for neutrality tests.

For the western Tianshan Mountains population, the highest genetic diversity was found in the YLK population (Hd = 0.963, *π* = 0.00398) from the natural forests, while the lowest (Hd = 0.591, *π* = 0.00172) was found in the YLN population from plantations. Overall, the genetic diversity of natural forest populations is slightly higher than that of plantation populations (Table 3).

### Genetic Differentiation and Genetic Flow

The pairwise *F*_*st*_ values for genetic differentiation varied from −0.0866 to 0.9428, based on the genes combined. The maximum value was detected between the HM and FX populations, and the minimum value was detected between the JZ and YLB populations. Five populations (CF, CY, FX, HM, and PL) exhibited significant genetic differentiation from other populations. The SY population showed higher genetic differences with other populations, whereas there was a certain gene flow between the BY population and the JZ, YLB, YLK, and YLZS populations, respectively. Additionally, no genetic differentiation was observed between the JZ population and the YL group. The *N*_*m*_ values indicated that frequent genetic flow occurred between the YL group and the JZ population (Table 4).

**TABLE 4:** Genetic differentiation (*F*_*st*_) and gene flow (*N*_*m*_) among 17 populations of *A. mali* based on combined genes.

Population code	BY	CF	CY	FX	HM	JZ	PL	SY	YLB	YLH	YLK	YLN	YLQ	YLT	YLZ	YLZH	YLZS
**BY**	—	0.12	0.40	0.11	0.09	1.74	0.20	0.64	**1.63**	0.66	**1.35**	0.80	0.68	0.99	0.86	0.81	**1.58**
**CF**	**0.6834**	—	0.27	0.06	0.06	0.09	0.10	0.07	0.10	0.07	0.09	0.07	0.08	0.08	0.08	0.10	0.10
**CY**	**0.3859**	**0.4816**	—	0.18	0.13	0.29	0.28	0.21	0.31	0.21	0.28	0.20	0.24	0.22	0.22	0.32	0.34
**FX**	**0.6915**	**0.7955**	**0.5819**	—	0.02	0.07	0.31	0.03	0.07	0.04	0.06	0.03	0.05	0.04	0.04	0.07	0.08
**HM**	**0.7346**	**0.8084**	**0.6524**	**0.9428**	—	0.06	0.08	0.03	0.06	0.03	0.06	0.03	0.04	0.04	0.04	0.06	0.07
**JZ**	0.1258	**0.7272**	**0.4606**	**0.7911**	**0.8102**	—	0.14	**1.02**	−3.14	**5.32**	−5.30	**47.97**	**4.53**	**30.04**	**9.96**	**6.78**	−4.98
**PL**	**0.5544**	**0.7226**	**0.4747**	**0.4483**	**0.7686**	**0.6473**	—	0.08	0.14	0.08	0.13	0.08	0.10	0.09	0.09	0.15	0.16
**SY**	**0.2824**	**0.7826**	**0.5443**	**0.8812**	**0.8930**	0.1973	**0.7519**	—	0.92	0.52	0.90	0.60	0.58	0.72	0.71	0.47	0.74
**YLB**	0.1327	**0.7192**	**0.4502**	**0.7796**	**0.8003**	−0.0866	**0.6358**	0.2130	—	**4.22**	−5.03	**4.66**	**4.98**	**8.65**	**6.61**	**12.84**	−5.21
**YLH**	**0.2750**	**0.7740**	**0.5381**	**0.8746**	**0.8871**	0.0449	**0.7504**	**0.3260**	0.0560	—	**6.33**	**11.44**	−6.59	**3.23**	−6.00	**1.30**	**1.32**
**YLK**	0.1563	**0.7267**	**0.4697**	**0.7937**	**0.8112**	−0.0495	**0.6576**	0.2171	−0.0523	0.0380	—	**6.28**	**6.53**	−31.36	−132.84	**2.71**	**33.17**
**YLN**	0.2373	**0.7876**	**0.5534**	**0.8966**	**0.9076**	0.0052	**0.7687**	**0.2936**	0.0509	0.0214	0.0383	—	**2.20**	-17.85	-8.40	0.74	**1.45**
**YLQ**	**0.2683**	**0.7539**	**0.5101**	**0.8413**	**0.8569**	0.0523	**0.7155**	**0.3028**	0.0478	−0.0394	0.0369	0.1019	—	**1.89**	**38.63**	**2.47**	**1.64**
**YLT**	0.2019	**0.7675**	**0.5301**	**0.8618**	**0.8738**	0.0083	**0.7334**	**0.2587**	0.0281	0.0718	-0.0080	-0.0142	0.1168	—	−11.09	0.77	**1.88**
**YLZ**	0.2258	**0.7607**	**0.5266**	**0.8477**	**0.8596**	0.0245	**0.7258**	**0.2604**	0.0364	−0.0435	−0.0019	−0.0307	0.0064	−0.0231	—	**1.02**	**1.55**
**YLZH**	0.2354	**0.7140**	**0.4353**	**0.7775**	**0.8051**	0.0356	**0.6304**	**0.3493**	0.0191	0.1614	0.0845	**0.2521**	0.0919	0.2448	0.1963	—	**31.68**
**YLZS**	0.1368	**0.7060**	**0.4271**	**0.7568**	**0.7834**	−0.0529	**0.6042**	**0.2513**	−0.0504	0.1588	0.0075	0.1475	0.1323	0.1173	0.1390	0.0078	—

For population code information, Table 1. F_st_ values are below the diagonal and N_m_ values are above the diagonal. Bold F_st_ values and N_m_ values indicate great genetic differentiation and frequent gene flow between populations (F_st_ > 0.25, or N_m_ > 1), respectively.

**FIGURE 2:**
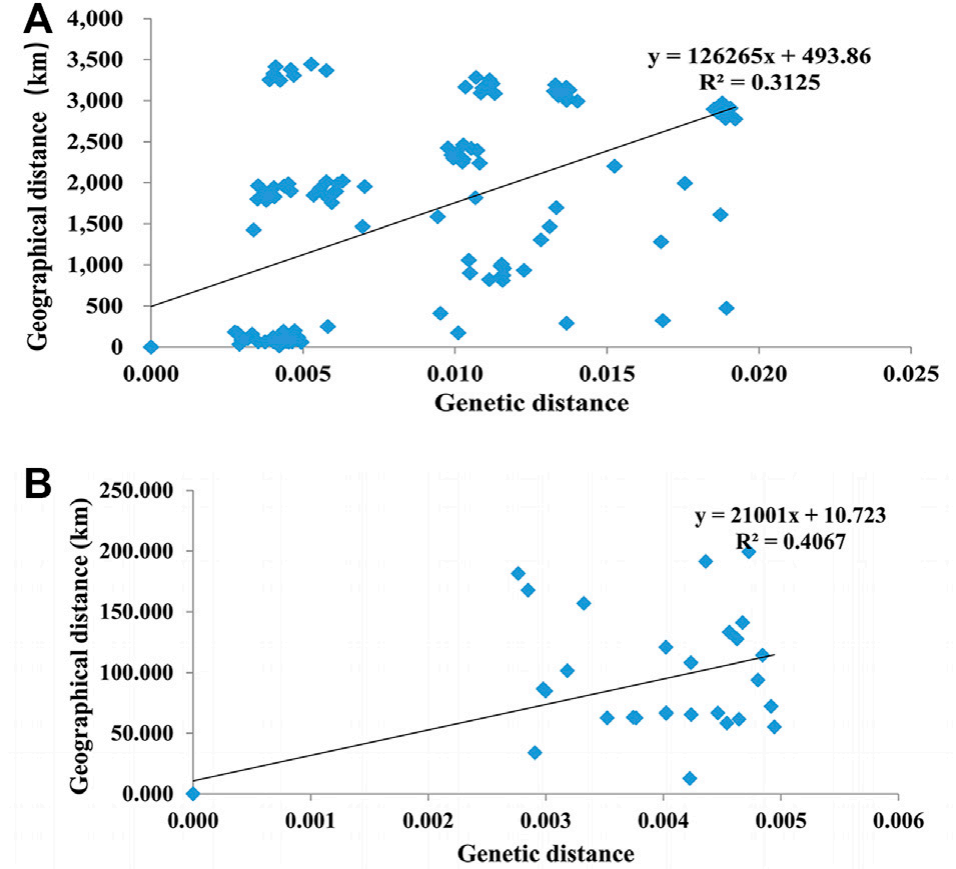
The correlation between genetic distance and geographical distance among 17 populations in China **(A)** and nine populations in the Western Tianshan Mountains **(B)** of *A. mali*.

AMOVA showed that overall variation mainly derived from variation among populations (53.25% among 17 populations and 63.09% among nine groups), and genetic variation within populations was slightly lower (Table 5). The Mantel test revealed a significant positive relationship for all populations (R = 0.5590 and *p* = 0.0001) (Figure 2A) while no significant correlation for the western Tianshan Mountains populations (R = 0.6377, *p* = 0.952) (Figure 2B) between the average pairwise genetic distance and geographic distance, indicating that genetic differentiation in populations is probably caused by geographical isolation.

**TABLE 5:** Analysis of molecular variance (AMOVA) based on combined genes.

Source of variation	d. f	Sum of squares	Variance components	Percentage of variation
**17 populations**				
Among populations	16	1,239.740	6.02927 Va	53.25
Within populations	188	995.318	5.29425 Vb	46.75
Total	204	2,235.059	11.32352	—
**9 groups**				
Among populations	8	1,163.385	9.34708 Va	63.09
Within populations	196	1,071.673	5.46772 Vb	36.91
Total	204	2,235.059	14.81480	—

a Va, Vb: Number of variance components.

### Phylogenetic Analysis and Network Construction

**FIGURE 3:**
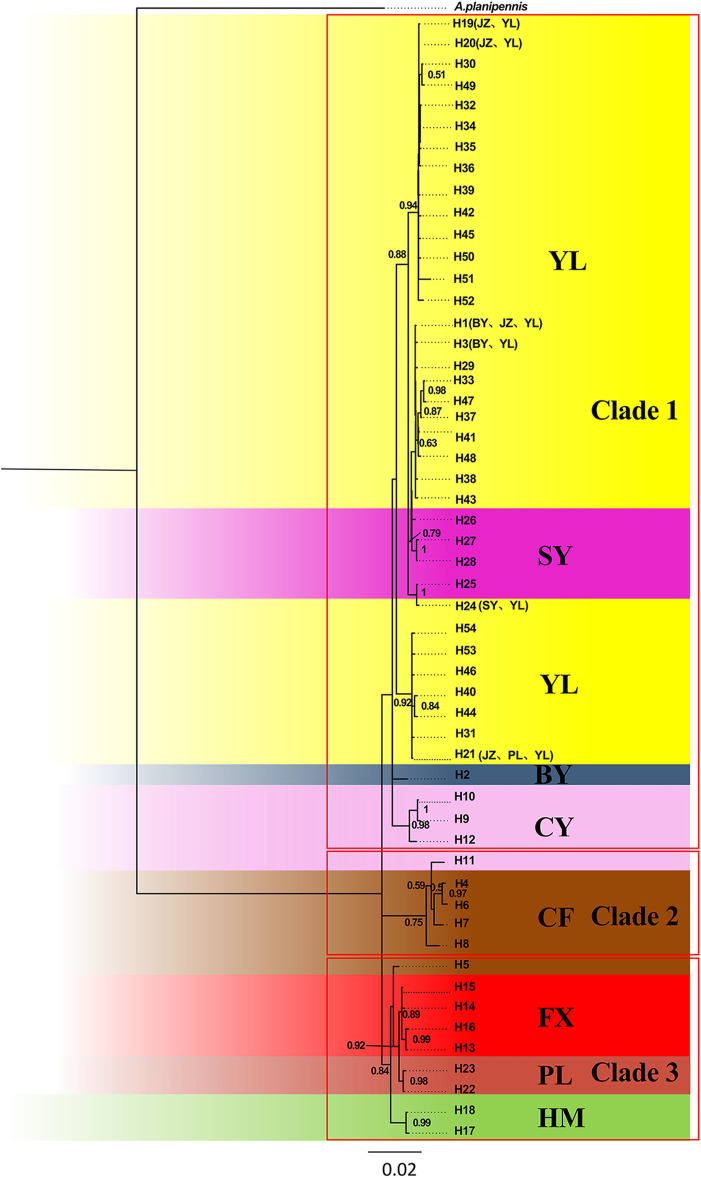
Bayesian inference (BI) phylogenetic tree inferred from haplotypes based on combined genes.

Based on the combined genes, the BI phylogenetic trees displayed three parallel clades: Clade 1 containing YL, JZ, SY, BY (these four populations formed the biggest subdivision), and CY populations (another subdivision); Clade 2 containing CF; Clade 3 containing CF, FX, HM, and PL (Figure 3). Within Clade 1, the biggest subdivision was divided into three clusters. Two of them contained pure YL haplotypes despite sharing several haplotypes with other populations, while the other was mixed with SY population.

**FIGURE 4:**
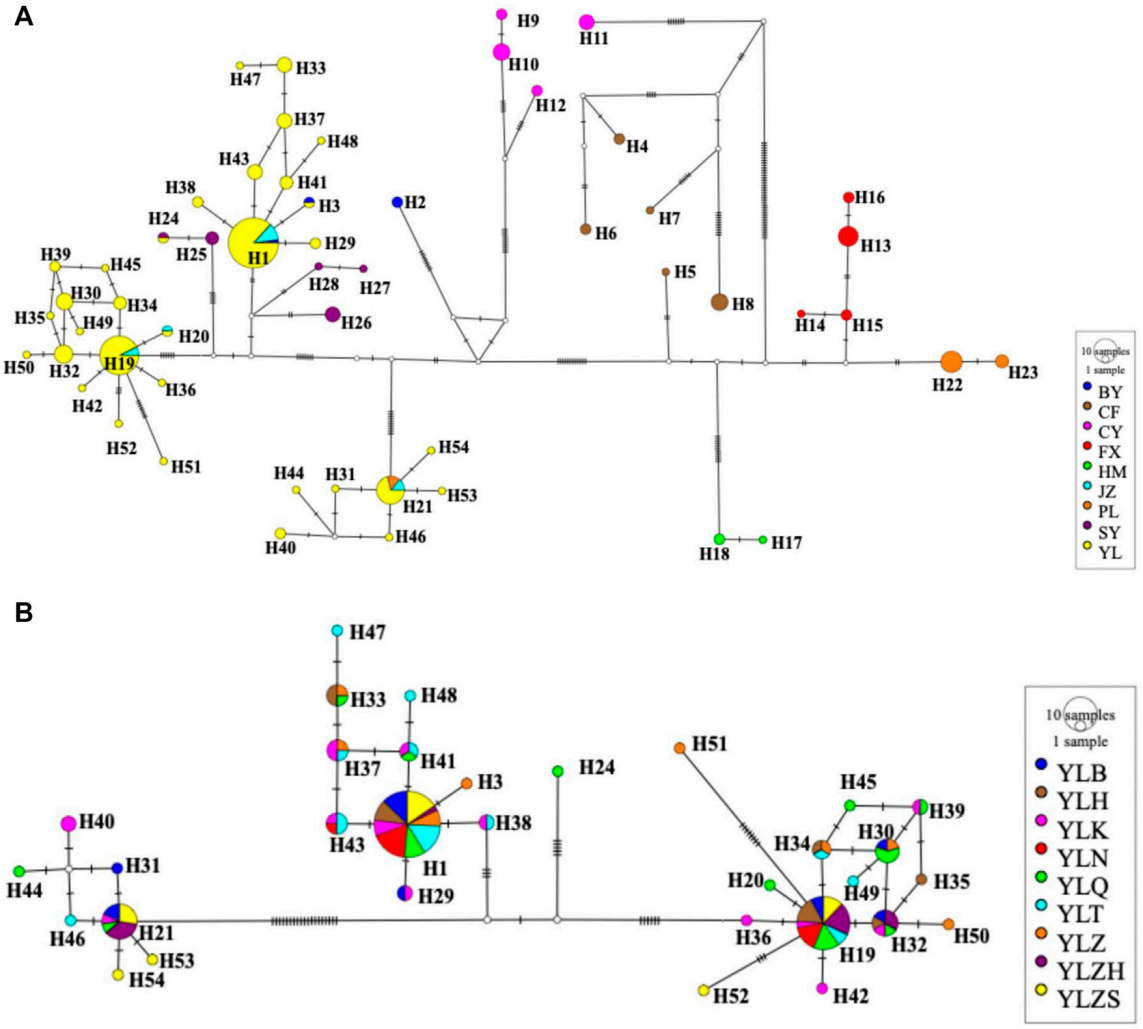
Median-joining haplotype networks of 17 different populations **(A)** and the YL group **(B)** of *A. mali* based on concatenated genes. Colored circles represent different haplotypes, solid lines between haplotypes represent mutation steps. The area of the circle is proportional to the number of haplotypes.

Median-joining networks inferred from the combined genes also supported similar phylogenetic relationships. Based on combined genes, the median-joining haplotype network for all populations did not exhibit the ancestral haplotype of *A. mali* across overall populations (Figure 4A), whereas three ancestral haplotypes (H1, H19, and H21) for the network of the YL group were found (Figure 4B). These three ancestral haplotypes accounted for more than half (73/129) of the individuals in the YL group. Furthermore, the ancestral haplotypes of the YL group were more apparent in single genes, namely H1, H16, and H18 in *COI*; H1, H13, and H14 in *COII*; and H1, H14, and H15 in *CytB*. In contrast, the median-joining network of the haplotypes based on a single gene of *COII* was different from the results obtained using *COI*, *CytB*, and combined genes (Figure 4; Supplementary Figure S1), which resulted from a lower nucleotide diversity and less genetic variation caused by the shorter *COII* sequence.

### Demographic History

For the neutral test, despite one significantly positive Tajima’s D value in the YLH population (2.38509, *p* < 0.05), most Tajima’s D and Fu’s Fs values (Table 3) in the overall population displayed no significance, suggesting that the Chinese population, including the Tianshan population from natural forests, did not display population expansion (Rogers and Harpending, 1992; Harpending et al., 1998).

**FIGURE 5:**
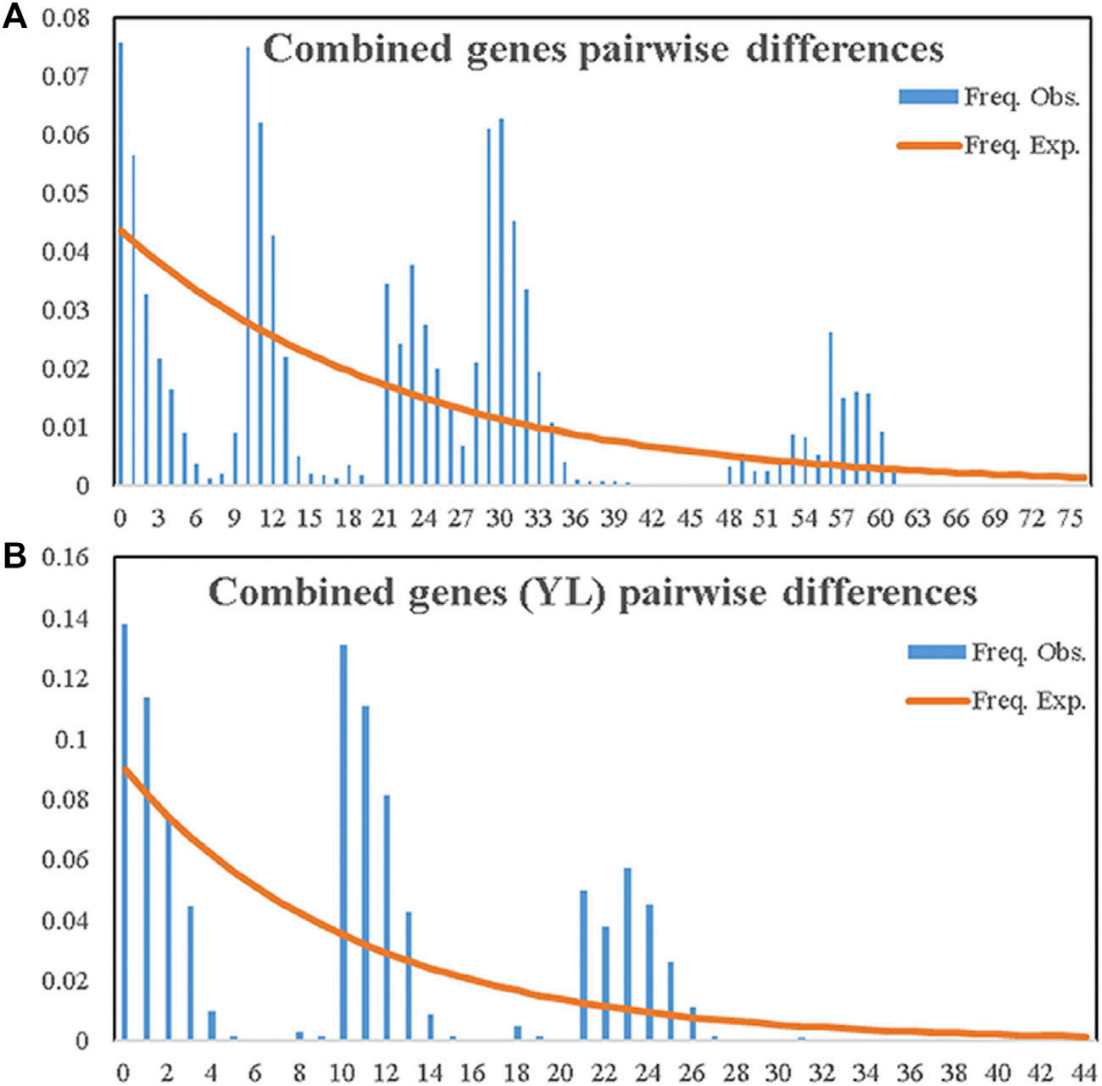
Observed and expected mismatch distribution analyses of entire samples of *A. mali* in China **(A)** and the YL group **(B)** based on combined genes.

Distributions of pairwise differences obtained with either single or combined genetic data across populations exhibited declined simulated curves (Figure 5A; Supplementary Figure S2). This suggested that the demographic history of *A. mali* populations in China fitted equilibrium models and that these insects did not experience recent population expansion. The same result was also obtained among Tianshan populations (Figure 5B).

## Discussion

mtDNA has been widely employed to investigate intraspecific variation in invertebrates because of its lack of recombination, maternal inheritance, conserved structure, higher mutation rate, and relatively high evolutionary rate. In this study, we report the genetic diversity and structure of 205 individuals from 17 *A. mali* populations sampled throughout their main distribution areas in China. This study was based on the complete sequences of three mitochondrial genes, *COI*, *COII*, and *CytB*, in contrast to the commonly employed partial gene sequences. Our analysis of the three mitochondrial genes revealed considerably high genetic diversity in *A. mali* populations in the sampled areas.

The beetle was reported to invade the Tianshan Wild Fruits Forests when its host apple tree was introduced from the Shandong province (Ji et al., 2004; Bozorov et al., 2019). However, it was not been found within the province during our project, with a 5-years sampling period, neither by investigating nor through yellow sticky card traps in the fields of apple orchards in different seasons.

Furthermore, older research recorded the presence of this beetle with wider ranges of distribution in China (Chen and Yao, 1997; Xiang, 1997; Li, 1998; Qu et al., 1998; Wang et al., 1998). However, these distributions have not been corroborated. The reason for this may be that the old host apple trees were cut, and new seedlings were quarantined strictly, causing the disappearance of some populations.

Hd and π are important indicators of genetic diversity. Here, overall populations had high haplotype diversity (from 0.590 to 0.963); however, most of them displayed moderate or low nucleotide diversity (<0.005). Grant and Bowen (1998) defined the genetic variability of populations into four categories based on mtDNA markers: i) low Hd and low *π*, ii) high Hd and low *π*, iii) low Hd and high *π*, and iv) high Hd and high *π*. Most *A. mali* populations fitted the second category, excluding BY, CF, and CY. According to Grant and Bowen (1998), the situation was attributed to population expansion after a period of low effective population size. However, this explanation seemed incompletely suitable for *A. mali*. based on our distributions of pairwise differences and neutral test data, which supported that the YL population did not experience recent population expansion. The inference was also not seemly obtained in another previous study (Ingman et al., 2000), and many species fell into the second category (Grant and Bowen, 1998). Yet, for overall populations, the levels of haplotype diversity and nucleotide diversity fall into the fourth class (high Hd and high *π*), which was considered as secondary contacts between previously differentiated allopatric lineages, or attributable to a long evolutionary history in a large stable population (Grant and Bowen,1998).

For the western Tianshan populations, genetic diversity in plantations was slightly reduced compared to that in natural forests. Natural forest populations showed more haplotypes than those seen in plantation populations, even with a similar sampling size (Tables 1, 3). Considering low π in each population, the national Natural Reserve population YLK displayed the highest genetic diversity (Hd = 0.963, *π* = 0.00398), followed by another natural forest site population, YLZ (Hd = 0.933, *π* = 0.00294). Those two sites represented a typically dense growth region of the wild apple tree (Zhou et al., 2020), indicating that it is possible for the population to have shifted from natural forests to plantations, at least in Tianshan Mountains. This is seemingly suitable for the above hypothesis.

Phylogeny relationships revealed that the YL and CY populations occupied one of three clear clades, in which three subdivisions could identified, corresponding to three star-liking clusters caused by three ancestral haplotypes, suggesting that YL likely has a long population history. Additionally, although the SY population was included in this clade, we could identify, through haplotype networks, the presence of many mutation steps between the haplotypes of the SY population and the ancestral haplotypes H1 and H19 of the YL group. These occurred a certain degree of genetic differentiation, which was supported by *Fst* values (Table 4).

The high level of genetic differentiation among all populations of *A. mali* indicated that the geographic isolation of insects is one of the important factors for genetic differentiation in different populations (Forbes et al., 2017; Goodman et al., 2019), which was supported by the positive correlation between genetic distance and geographic distance (Figure 2), while the geographic isolation was hard to be observed for the recent invasive pests (Yao et al., 2020). Moreover, the major proportion of molecular genetic variation existed among populations (Table 5). Notably, a larger proportion of molecular genetic variation (63.09%) was present between the YL population and the other eight populations. Similar genetic differentiation results for *A. mali* were also reported in a study by Fang et al. (2017), which could also demonstrate that the beetle in Tianshan was not recently introduced.

The population genetic differentiation of a species is not only affected by distance and geographic isolation but also its own biological characteristics and environmental conditions, such as human factors, flight capacity, population size, and habitat specialization (Sumner et al., 2004; Fuentes-Contreras et al., 2008; Du et al., 2020). Species with high dispersal ability and a large number of individuals in the population can maintain gene flow and pan-mixing. In contrast, low dispersal ability and poor habitat availability cause species subgroups to become isolated, reducing gene flow (Louy et al., 2007; Dixo et al., 2009). *A. mali* is generally regarded as a sedentary species with a certain natural diffusion ability of up to 35 m (Ma et al., 2021). The weak flight capacity of *A. mali* can reduce gene flow among populations.

For sedentary insects such as *A. mali*, anthropic movement of infected wood or wood products provides routes for the hitchhiking of live adults and larvae, which may increase gene exchange between populations. The findings of this study also suggest that frequent gene flow between natural forests and plantation populations, which may be caused by human activities, possibly enhanced the adaptability of *A. mali* along the western Tianshan Mountains and resulted in frequent outbreaks (Chapuis et al., 2011; Xu et al., 2019). Therefore, we assume that human factors caused the continued outbreak of *A. mali* in the western Tianshan Mountains.

Considering the outbreaks of the beetle, present in both natural forests and in plantations the Tianshan Mountains, we think that the decline in host forests was the main factor causing the rapid growth in beetle population size, because *A. mali* is a secondary pest and attacks the weak host tree. In the last few decades, the wild fruit forest in the western Tianshan Mountains gradually declined, probably caused by climatic and environmental factors, which provided a chance for the beetle outbreak. Subsequently, low plant diversity in the undergrowth made the natural enemies of the beetle lose their substitute host, leading to a reduction in population size. For the feeding of livestock, local people had to mow the grass growing in the wild fruit forest. Finally, poor orchard management also facilitated the rapid growth of the beetle population.

## Data Availability

The datasets presented in this study can be found in online repositories. The names of the repository/repositories and accession number(s) can be found in the article/Supplementary Material.

## Supplementary Material

Supplementary Figure S1 Median-joining haplotype networks of 17 different populations of *A. mali* based on *COI* **(A)**, *COII* **(B)** and *CytB* **(C)**. Colored circles represent different haplotypes, solid lines between haplotypes represent mutation steps. The area of the circle is proportional to the number of haplotypes.

Supplementary Figure S2 Observed and expected mismatch distribution analyses of entire samples of *A. mali* in China based on *COI*, *COII* and *CytB* genes.

Supplementary Table S1 PCR primers designed for the *COI*, *COII*, and *CytB* genes of *A. mali*.

Supplementary Table S2 Genetic diversity parameters among 17 populations of *A. mali* based on *COI*, *COII*, and *CytB* genes.

Supplementary Table S3 Haplotype distribution of mitochondrial the *COI* gene of *A. mali* in each population.

Supplementary Table S4 Haplotype distribution of mitochondrial the *COII* gene of *A. mali* in each population.

Supplementary Table S5 Haplotype distribution of mitochondrial the *CytB* gene of *A. mali* in each population.

Supplementary Table S6 Haplotype distribution of combined mitochondrial genes of *A. mali* in each population.
