# Supplementary material for: Population Genetics Reveals That the Western Tianshan Mountains Populations of Agrilus mali (Coleoptera: Buprestidae) May Have Not been Recently Introduced
Source: Front Genet. 2022 Mar 24;13:857866. doi: 10.3389/fgene.2022.857866 (PMC8988243; doi:10.3389/fgene.2022.857866)
Supplement: Supplementary file 1 [file Table1.DOCX]

**Table S1. Special PCR primers aimed at *COI, COII* and *CytB* genes of *A.mali*.**

| Gene | Name | Primer Sequences (5’-3’) | Tm(℃) |
| --- | --- | --- | --- |
| *COI* | *COI*-F | ATTATGAACAAGTGATTATTTTCA | 46 |
|  | *COI*-R | AATTATATAAAATAGGTAATTCAGAAT |  |
| *COII* | *COII*-F | TCCCACAACATTTCTTAG | 49 |
|  | *COII*-R | CTTGCTTTCAGCCATCTA |  |
| *CytB* | *CytB*-F | TATAAGACAACCAGGCCAGT | 53 |
|  | *CytB*-R | TTTGCCCCGTTATCGTTATG |  |
