# Supplementary figures and images for: Population Genetics Reveals That the Western Tianshan Mountains Populations of Agrilus mali (Coleoptera: Buprestidae) May Have Not been Recently Introduced
Source: Front Genet. 2022 Mar 24;13:857866. doi: 10.3389/fgene.2022.857866 (PMC8988243; doi:10.3389/fgene.2022.857866)

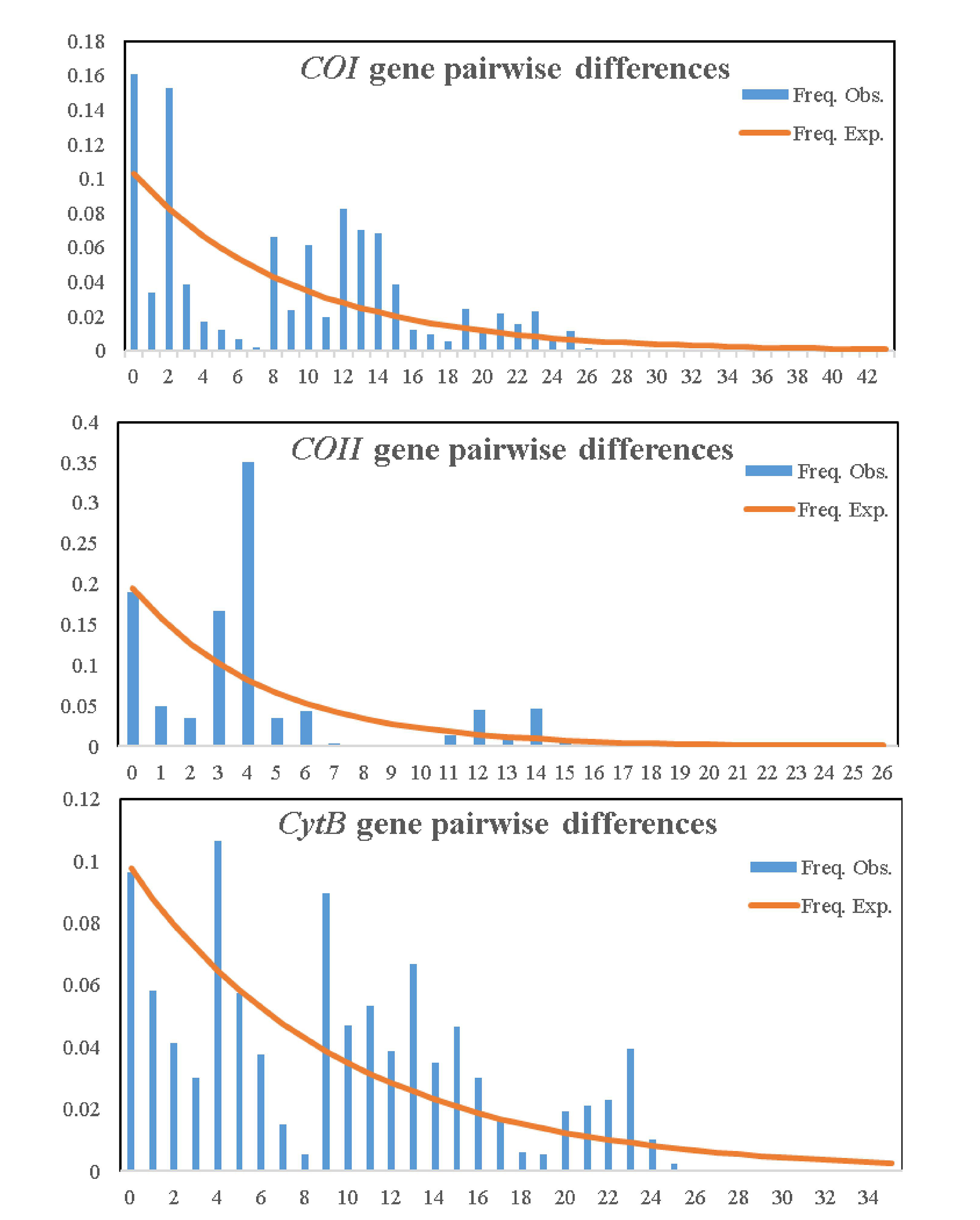

Supplement: Supplementary file 2 [file Image2.TIF]

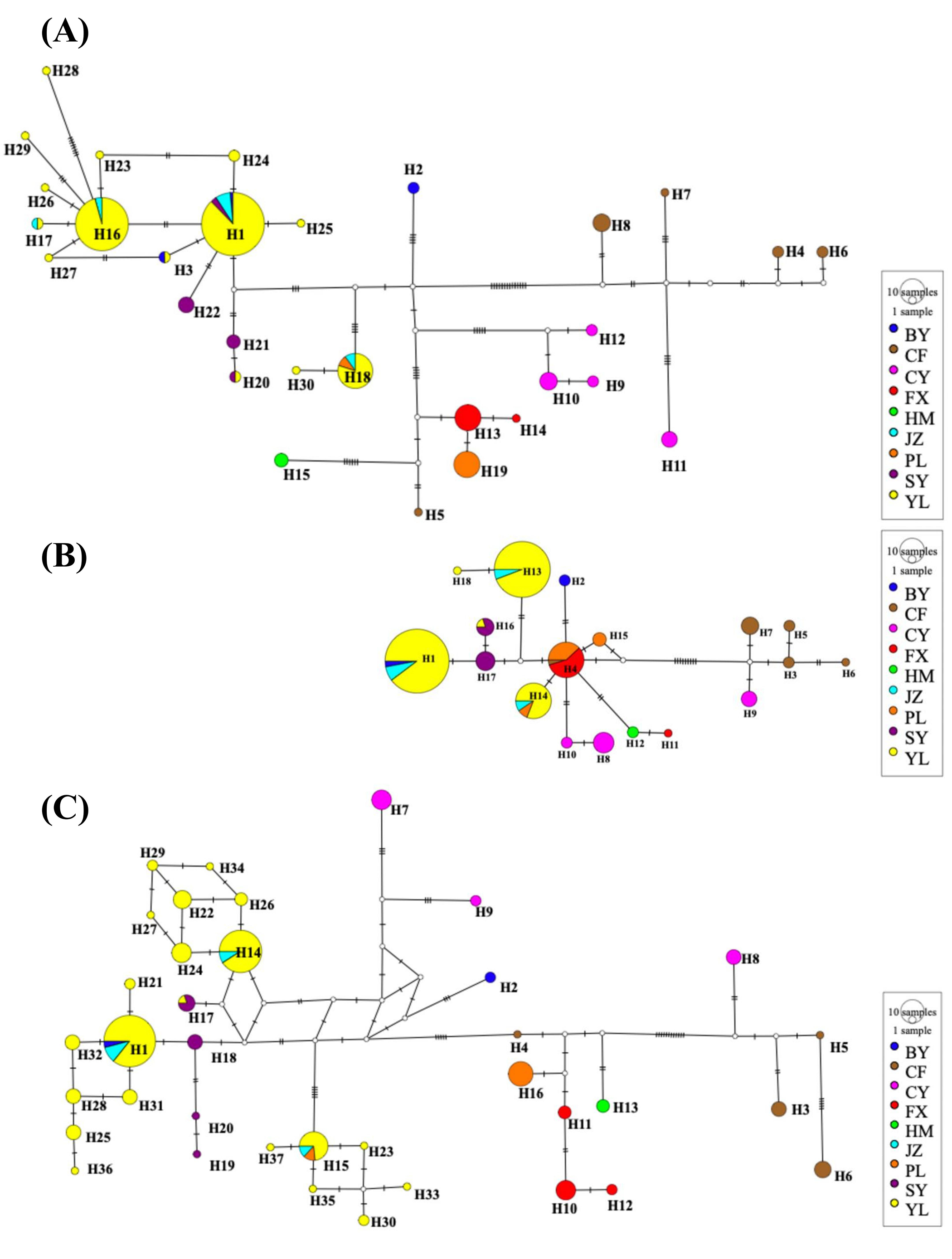

Supplement: Supplementary file 3 [file Image1.TIF]
