# Supplementary material for: Population Genetics Reveals That the Western Tianshan Mountains Populations of Agrilus mali (Coleoptera: Buprestidae) May Have Not been Recently Introduced
Source: Front Genet. 2022 Mar 24;13:857866. doi: 10.3389/fgene.2022.857866 (PMC8988243; doi:10.3389/fgene.2022.857866)
Supplement: Supplementary file 4 [file Table2.DOCX]

**Table. S2. Genetic diversity parameters among 17 populations of *A.mali* based on *COI, COII* and *CytB* genes**

| Sample | *COI* | | | | | *COII* | | | | | *CytB* | | | | |
| --- | --- | --- | --- | --- | --- | --- | --- | --- | --- | --- | --- | --- | --- | --- | --- |
| Code | **S** | **h** | **Hd** | **K** | **π** | **S** | **h** | **Hd** | **K** | **π** | **S** | **h** | **Hd** | **K** | **π** |
| BY | 10 | 3 | 0.833 | 6.5 | 0.00466 | 5 | 2 | 0.667 | 3.333 | 0.00573 | 9 | 2 | 0.667 | 6.000 | 0.00641 |
| CF | 29 | 5 | 0.782 | 8.182 | 0.00587 | 14 | 5 | 0.782 | 3.564 | 0.00612 | 21 | 4 | 0.709 | 6.109 | 0.00653 |
| CY | 26 | 4 | 0.769 | 11.462 | 0.00822 | 14 | 3 | 0.641 | 6.538 | 0.01123 | 25 | 3 | 0.641 | 10.974 | 0.01172 |
| FX | 1 | 2 | 0.167 | 0.167 | 0.00012 | 0 | 1 | 0 | 0 | 0 | 3 | 3 | 0.621 | 1.121 | 0.00120 |
| HM | 0 | 1 | 0 | 0 | 0 | 1 | 2 | 0.667 | 0.667 | 0.00115 | 0 | 1 | 0 | 0 | 0 |
| JZ | 11 | 4 | 0.733 | 3.978 | 0.00285 | 6 | 3 | 0.689 | 2.756 | 0.00473 | 11 | 3 | 0.689 | 4.533 | 0.00484 |
| PL | 13 | 2 | 0.282 | 3.667 | 0.00263 | 2 | 3 | 0.590 | 0.667 | 0.00115 | 14 | 2 | 0.282 | 3.949 | 0.00422 |
| SY | 6 | 4 | 0.778 | 2.867 | 0.00205 | 1 | 2 | 0.533 | 0.533 | 0.00092 | 6 | 4 | 0.733 | 2.511 | 0.00268 |
| YLB | 10 | 3 | 0.692 | 4.000 | 0.00287 | 6 | 3 | 0.692 | 2.769 | 0.00476 | 14 | 7 | 0.846 | 5.385 | 0.00575 |
| YLH | 2 | 2 | 0.538 | 1.077 | 0.00077 | 4 | 2 | 0.538 | 2.154 | 0.00370 | 7 | 6 | 0.833 | 3.385 | 0.00362 |
| YLK | 11 | 5 | 0.728 | 3.574 | 0.00256 | 7 | 4 | 0.669 | 2.676 | 0.00460 | 14 | 10 | 0.919 | 5.338 | 0.00575 |
| YLN | 2 | 2 | 0.485 | 0.970 | 0.00070 | 4 | 2 | 0.485 | 1.939 | 0.00333 | 5 | 3 | 0.591 | 2.106 | 0.00225 |
| YLQ | 14 | 5 | 0.649 | 2.386 | 0.00171 | 7 | 4 | 0.591 | 2.292 | 0.00394 | 15 | 10 | 0.889 | 4.117 | 0.00440 |
| YLT | 13 | 6 | 0.621 | 1.954 | 0.00140 | 6 | 3 | 0.451 | 1.804 | 0.00310 | 15 | 9 | 0.843 | 3.510 | 0.00375 |
| YLZ | 11 | 5 | 0.756 | 3.022 | 0.00217 | 4 | 2 | 0.533 | 2.133 | 0.00367 | 7 | 7 | 0.867 | 3.400 | 0.00363 |
| YLZH | 10 | 3 | 0.591 | 4.939 | 0.00354 | 6 | 3 | 0.591 | 2.364 | 0.00406 | 12 | 4 | 0.742 | 5.091 | 0.00544 |
| YLZS | 13 | 5 | 0.771 | 5.162 | 0.00370 | 6 | 3 | 0.705 | 2.819 | 0.00484 | 12 | 4 | 0.743 | 5.333 | 0.00570 |
| Totol | 71 | 30 | 0.834 | 8.576 | 0.00615 | 26 | 18 | 0.804 | 4.098 | 0.00704 | 54 | 37 | 0.903 | 9.238 | 0.00987 |
