# Supplementary material for: Population Genetics Reveals That the Western Tianshan Mountains Populations of Agrilus mali (Coleoptera: Buprestidae) May Have Not been Recently Introduced
Source: Front Genet. 2022 Mar 24;13:857866. doi: 10.3389/fgene.2022.857866 (PMC8988243; doi:10.3389/fgene.2022.857866)
Supplement: Supplementary file 5 [file Table5.DOCX]

**Table S5. Haplotypes distribution of mitochondrial *CytB* gene of *A. mali* in each group**

|  | BY | CF | CY | FX | HM | JZ | PL | SY | YLB | YLH | YLK | YLN | YLQ | YLT | YLZ | YLZH | YLZS |
| --- | --- | --- | --- | --- | --- | --- | --- | --- | --- | --- | --- | --- | --- | --- | --- | --- | --- |
| H1 | 2 |  |  |  |  | 5 |  |  | 5 | 4 | 4 | 7 | 4 | 7 | 4 | 1 | 6 |
| H2 | 2 |  |  |  |  |  |  |  |  |  |  |  |  |  |  |  |  |
| H3 |  | 4 |  |  |  |  |  |  |  |  |  |  |  |  |  |  |  |
| H4 |  | 1 |  |  |  |  |  |  |  |  |  |  |  |  |  |  |  |
| H5 |  | 1 |  |  |  |  |  |  |  |  |  |  |  |  |  |  |  |
| H6 |  | 5 |  |  |  |  |  |  |  |  |  |  |  |  |  |  |  |
| H7 |  |  | 7 |  |  |  |  |  |  |  |  |  |  |  |  |  |  |
| H8 |  |  | 4 |  |  |  |  |  |  |  |  |  |  |  |  |  |  |
| H9 |  |  | 2 |  |  |  |  |  |  |  |  |  |  |  |  |  |  |
| H10 |  |  |  | 7 |  |  |  |  |  |  |  |  |  |  |  |  |  |
| H11 |  |  |  | 3 |  |  |  |  |  |  |  |  |  |  |  |  |  |
| H12 |  |  |  | 2 |  |  |  |  |  |  |  |  |  |  |  |  |  |
| H13 |  |  |  |  | 3 |  |  |  |  |  |  |  |  |  |  |  |  |
| H14 |  |  |  |  |  | 3 |  |  | 2 | 4 | 3 | 4 | 5 | 2 | 1 | 5 | 4 |
| H15 |  |  |  |  |  | 2 | 2 |  | 2 |  | 1 |  |  |  |  | 4 | 4 |
| H16 |  |  |  |  |  |  | 11 |  |  |  |  |  |  |  |  |  |  |
| H17 |  |  |  |  |  |  |  | 4 |  |  |  |  | 1 |  |  |  |  |
| H18 |  |  |  |  |  |  |  | 4 |  |  |  |  |  |  |  |  |  |
| H19 |  |  |  |  |  |  |  | 1 |  |  |  |  |  |  |  |  |  |
| H20 |  |  |  |  |  |  |  | 1 |  |  |  |  |  |  |  |  |  |
| H21 |  |  |  |  |  |  |  |  | 1 |  | 1 |  |  |  |  |  |  |
| H22 |  |  |  |  |  |  |  |  | 1 |  |  |  | 3 | 1 | 1 |  |  |
| H23 |  |  |  |  |  |  |  |  | 1 |  |  |  |  |  |  |  |  |
| H24 |  |  |  |  |  |  |  |  | 1 | 1 | 1 |  | 1 |  | 1 | 2 |  |
| H25 |  |  |  |  |  |  |  |  |  | 2 |  |  | 1 |  | 1 |  |  |
| H26 |  |  |  |  |  |  |  |  |  | 1 |  |  |  | 1 | 1 |  |  |
| H27 |  |  |  |  |  |  |  |  |  | 1 |  |  |  |  |  |  |  |
| H28 |  |  |  |  |  |  |  |  |  |  | 2 |  |  | 1 | 1 |  |  |
| H29 |  |  |  |  |  |  |  |  |  |  | 1 |  | 1 |  |  |  |  |
| H30 |  |  |  |  |  |  |  |  |  |  | 2 |  |  | 2 |  |  |  |
| H31 |  |  |  |  |  |  |  |  |  |  | 1 |  | 1 | 2 |  |  |  |
| H32 |  |  |  |  |  |  |  |  |  |  | 1 | 1 |  |  |  |  |  |
| H33 |  |  |  |  |  |  |  |  |  |  |  |  | 1 |  |  |  |  |
| H34 |  |  |  |  |  |  |  |  |  |  |  |  | 1 |  |  |  |  |
| H35 |  |  |  |  |  |  |  |  |  |  |  |  |  | 1 |  |  |  |
| H36 |  |  |  |  |  |  |  |  |  |  |  |  |  | 1 |  |  |  |
| H37 |  |  |  |  |  |  |  |  |  |  |  |  |  |  |  |  | 1 |
