# Supplementary material for: Population Genetics Reveals That the Western Tianshan Mountains Populations of Agrilus mali (Coleoptera: Buprestidae) May Have Not been Recently Introduced
Source: Front Genet. 2022 Mar 24;13:857866. doi: 10.3389/fgene.2022.857866 (PMC8988243; doi:10.3389/fgene.2022.857866)
Supplement: Supplementary file 6 [file Table3.DOCX]

**Table S3. Haplotypes distribution of mitochondrial *COI* gene of *A. mali* in each group**

|  | BY | CF | CY | FX | HM | JZ | PL | SY | YLB | YLH | YLK | YLN | YLQ | YLT | YLZ | YLZH | YLZS | GenBank accession numbers |
| --- | --- | --- | --- | --- | --- | --- | --- | --- | --- | --- | --- | --- | --- | --- | --- | --- | --- | --- |
| H1 | 1 |  |  |  |  | 5 |  | 2 | 6 | 6 | 8 | 8 | 6 | 11 | 5 | 1 | 6 | OM177028 |
| H2 | 2 |  |  |  |  |  |  |  |  |  |  |  |  |  |  |  |  | OM177029 |
| H3 | 1 |  |  |  |  |  |  |  |  |  |  |  |  |  | 1 |  |  | OM177030 |
| H4 |  | 2 |  |  |  |  |  |  |  |  |  |  |  |  |  |  |  | OM177031 |
| H5 |  | 1 |  |  |  |  |  |  |  |  |  |  |  |  |  |  |  | OM177032 |
| H6 |  | 2 |  |  |  |  |  |  |  |  |  |  |  |  |  |  |  | OM177033 |
| H7 |  | 1 |  |  |  |  |  |  |  |  |  |  |  |  |  |  |  | OM177034 |
| H8 |  | 5 |  |  |  |  |  |  |  |  |  |  |  |  |  |  |  | OM177035 |
| H9 |  |  | 2 |  |  |  |  |  |  |  |  |  |  |  |  |  |  | OM177036 |
| H10 |  |  | 5 |  |  |  |  |  |  |  |  |  |  |  |  |  |  | OM177037 |
| H11 |  |  | 4 |  |  |  |  |  |  |  |  |  |  |  |  |  |  | OM177038 |
| H12 |  |  | 2 |  |  |  |  |  |  |  |  |  |  |  |  |  |  | OM177039 |
| H13 |  |  |  | 11 |  |  |  |  |  |  |  |  |  |  |  |  |  | OM177040 |
| H14 |  |  |  | 1 |  |  |  |  |  |  |  |  |  |  |  |  |  | OM177041 |
| H15 |  |  |  |  | 3 |  |  |  |  |  |  |  |  |  |  |  |  | OM177042 |
| H16 |  |  |  |  |  | 2 |  |  | 4 | 7 | 4 | 4 | 10 | 3 | 2 | 7 | 3 | OM177043 |
| H17 |  |  |  |  |  | 1 |  |  |  |  |  |  | 1 |  |  |  |  | OM177044 |
| H18 |  |  |  |  |  | 2 | 2 |  | 3 |  | 3 |  | 1 | 1 |  | 4 | 4 | OM177045 |
| H19 |  |  |  |  |  |  | 11 |  |  |  |  |  |  |  |  |  |  | OM177046 |
| H20 |  |  |  |  |  |  |  | 1 |  |  |  |  | 1 |  |  |  |  | OM177047 |
| H21 |  |  |  |  |  |  |  | 3 |  |  |  |  |  |  |  |  |  | OM177048 |
| H22 |  |  |  |  |  |  |  | 4 |  |  |  |  |  |  |  |  |  | OM177049 |
| H23 |  |  |  |  |  |  |  |  |  |  | 1 |  |  |  |  |  |  | OM177050 |
| H24 |  |  |  |  |  |  |  |  |  |  | 1 |  |  | 1 |  |  |  | OM177051 |
| H25 |  |  |  |  |  |  |  |  |  |  |  |  |  | 1 |  |  |  | OM177052 |
| H26 |  |  |  |  |  |  |  |  |  |  |  |  |  | 1 |  |  |  | OM177053 |
| H27 |  |  |  |  |  |  |  |  |  |  |  |  |  |  | 1 |  |  | OM177054 |
| H28 |  |  |  |  |  |  |  |  |  |  |  |  |  |  | 1 |  |  | OM177055 |
| H29 |  |  |  |  |  |  |  |  |  |  |  |  |  |  |  |  | 1 | OM177056 |
| H30 |  |  |  |  |  |  |  |  |  |  |  |  |  |  |  |  | 1 | OM177057 |
