# Supplementary material for: Population Genetics Reveals That the Western Tianshan Mountains Populations of Agrilus mali (Coleoptera: Buprestidae) May Have Not been Recently Introduced
Source: Front Genet. 2022 Mar 24;13:857866. doi: 10.3389/fgene.2022.857866 (PMC8988243; doi:10.3389/fgene.2022.857866)
Supplement: Supplementary file 7 [file Table4.DOCX]

**Table S4. Haplotypes distribution of mitochondrial *COII* gene of *A. mali* in each group**

|  | BY | CF | CY | FX | HM | JZ | PL | SY | YLB | YLH | YLK | YLN | YLQ | YLT | YLZ | YLZH | YLZS | GenBank accession numbers |
| --- | --- | --- | --- | --- | --- | --- | --- | --- | --- | --- | --- | --- | --- | --- | --- | --- | --- | --- |
| H1 | 2 |  |  |  |  | 5 |  |  | 6 | 6 | 9 | 8 | 6 | 13 | 6 | 1 | 6 | OM212329 |
| H2 | 2 |  |  |  |  |  |  |  |  |  |  |  |  |  |  |  |  | OM212330 |
| H3 |  | 2 |  |  |  |  |  |  |  |  |  |  |  |  |  |  |  | OM212331 |
| H4 |  | 1 |  | 12 |  |  | 8 |  |  |  |  |  |  |  |  |  |  | OM212332 |
| H5 |  | 2 |  |  |  |  |  |  |  |  |  |  |  |  |  |  |  | OM212333 |
| H6 |  | 1 |  |  |  |  |  |  |  |  |  |  |  |  |  |  |  | OM212334 |
| H7 |  | 5 |  |  |  |  |  |  |  |  |  |  |  |  |  |  |  | OM212335 |
| H8 |  |  | 7 |  |  |  |  |  |  |  |  |  |  |  |  |  |  | OM212336 |
| H9 |  |  | 4 |  |  |  |  |  |  |  |  |  |  |  |  |  |  | OM212337 |
| H10 |  |  | 2 |  |  |  |  |  |  |  |  |  |  |  |  |  |  | OM212338 |
| H11 |  |  |  | 1 |  |  |  |  |  |  |  |  |  |  |  |  |  | OM212339 |
| H12 |  |  |  |  | 2 |  |  |  |  |  |  |  |  |  |  |  |  | OM212340 |
| H13 |  |  |  |  |  | 3 |  |  | 4 | 7 | 4 | 4 | 11 | 4 | 4 | 7 | 4 | OM212341 |
| H14 |  |  |  |  |  | 2 | 2 |  | 3 |  | 3 |  | 1 | 1 |  | 4 | 5 | OM212342 |
| H15 |  |  |  |  |  |  | 3 |  |  |  |  |  |  |  |  |  |  | OM212343 |
| H16 |  |  |  |  |  |  |  | 4 |  |  |  |  | 1 |  |  |  |  | OM212344 |
| H17 |  |  |  |  |  |  |  | 6 |  |  |  |  |  |  |  |  |  | OM212345 |
| H18 |  |  |  |  |  |  |  |  |  |  | 1 |  |  |  |  |  |  | OM212346 |
